# Outcomes of allogeneic transplant in patients with *DDX41* mutated myelodysplastic syndrome and acute myeloid leukemia

**DOI:** 10.1038/s41409-022-01776-6

**Published:** 2022-08-20

**Authors:** Anmol Baranwal, Ahmad Nanaa, David Viswanatha, Rong He, James Foran, Talha Badar, William J. Hogan, Mark R. Litzow, Mithun Vinod Shah, Mrinal M. Patnaik, Aref Al-Kali, Hassan B. Alkhateeb

**Affiliations:** 1 Division of Hematology, Department of Medicine, Mayo Clinic, Rochester, MN USA; 2 Division of Hematopathology, Department of Laboratory Medicine and Pathology, Mayo Clinic, Rochester, MN USA; 3 Division of Hematology-Oncology, Blood and Marrow Transplantation Program, Department of Medicine, Mayo Clinic, Jacksonville, FL USA

**Keywords:** Medical research, Haematological cancer

## To the Editor:

The DEAD-box helicase 41 (*DDX41*) gene, located at 5q35.3 locus [1], is involved in interactions with spliceosome proteins and development of innate immune response [2, 3]. *DDX41*-mutated acute myeloid leukemia (AML) and higher-grade myelodysplastic syndrome (MDS) are reported to have favorable outcomes [4–6]. In this study, we describe outcomes of patients with *DDX41*-mutated MDS/AML undergoing allogeneic stem cell transplant (alloSCT).

We retrospectively reviewed patients with *DDX41*-mutated, WHO defined, MDS or AML [7]. The study was approved by Mayo Clinic Institutional Review Board. Patients with MDS/AML, found to have a *DDX41* mutation on Next Generation Sequencing performed on peripheral blood or bone marrow aspirate, per institutional policy, were included. Germline testing was not performed. We reviewed the Mayo Clinic electronic medical records to determine patient demographics, transplant characteristics, and post-transplant outcomes. Disease risk was determined using the revised International Prognostic Scoring System for MDS and the European Leukemia Network 2017 risk stratification for AML [8, 9].

Primary objective was to assess the effect of alloSCT on overall survival (OS). Secondary objectives included assessment of cumulative incidence of relapse and non-relapse mortality (NRM) post-transplant.

Patient and transplant characteristics were summarized using descriptive statistics. Kaplan–Meier and log-rank tests were used to estimate OS and compare time to disease progression/relapse. We used Cox proportional hazards for time dependent variable to determine the effect of alloSCT on OS [10]. Post-transplant NRM was calculated using competing risk analysis. R 4.1.1 (R Foundation for Statistical Computing) was used to perform all the statistical analyses.

Twenty-nine patients (21 (72.4%) males) were found to harbor *DDX41* mutation. Median age at diagnosis was 67 years (range: 50–82 years, Supplementary Table 1).

Twenty-two patients (75.9%) had a family history of cancer; 10 (34.5%) had family history of solid tumor, 7 (24.1%) had family history of hematologic malignancy, while 5 (17.2%) patients had family history of both solid tumors and hematologic malignancies. *DDX41* mutation was detected at a median variant allele frequency (VAF) of 46% (range 6–52%) (Supplementary Table 2). A known pathogenic *DDX41* mutation with VAF ≥ 40% was found in 20 (68.96%) patients, while 8 (27.59%) patients had a pathogenic DDX41 mutation with VAF < 40%. One patient had a *DDX41* variant of unknown significance only.

Nine (31%) patients had AML and 20 (69%) had MDS. Median follow-up since diagnosis was 21 months (range 1.5 months-12 years). Among patients with MDS, four (20%) had high-risk disease, 13 (65%) had intermediate-risk disease and three (15%) had low-risk disease. Of the 20 patients with MDS, three (15%) had MDS-EB1 and 13 (65%) had MDS-EB2. All nine patients with AML had intermediate-risk disease. Seven patients (24.7%) died during the follow-up period. Median OS for the entire cohort was 11.4 years with 5-year OS rate of 68.5%. OS did not differ between patients with MDS and AML (median 11.4 years vs NA, *P* = 0.5). Among patients undergoing alloSCT, the median time to disease progression before alloSCT was similar to those who did not undergo alloSCT (3.1 vs. 2.3 years, *P* = 0.96), suggesting that patients in both alloSCT and non-alloSCT groups had comparable rates of disease progression.

**Fig. 1:**
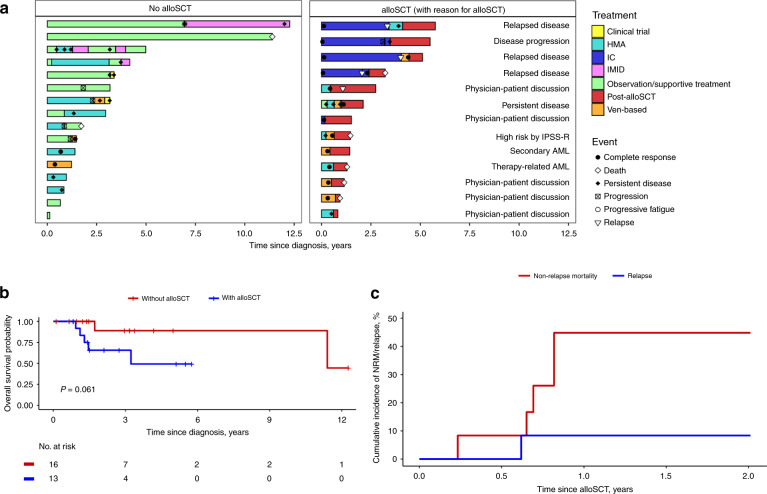
Clinical course, survival outcomes and post-transplant non-relapse mortality in patients with *DDX41* mutated MDS or AML. **a** Swimmer’s plot showing clinical course of patients with or without alloSCT. Among the non-alloSCT cohort, 11 (68.75%) patients with persistent/progressive disease were alive at last follow-up. In contrast, all five patients who died after alloSCT were in complete remission before alloSCT. **b** Overall survival since diagnosis comparing patients undergoing alloSCT to those without alloSCT. **c** Cumulative incidence of non-relapse mortality (NRM) and relapse. Abbreviations: HMA hypomethylating agent, IC intensive chemotherapy, IMID immunomodulator.

Thirteen patients (44.8%) (seven (53.8%) with AML and six (46.2%) with MDS) underwent alloSCT at a median age of 64 years (range 54–71 years). Eight (61.54%) patients had high-risk disease either by IPSS-R, relapse/persistent disease after therapy, or being considered therapy-related or secondary myeloid neoplasm (Fig. 1a). Five (38.46%) patients with intermediate-risk disease underwent alloSCT after patient-physician discussion.

Among patients with MDS, one (16.7%) had high-risk disease, four had intermediate-risk (66.7%) and one patient (16.7%) had low-risk disease. One patient with intermediate-risk MDS had transformed to AML before alloSCT. Four (30.8%) patients had persistent disease at the time of transplant. Nine (69.2%) patients were in complete morphological remission; two of whom (15.4%) had positive minimal residual disease testing. Of the 13 patients, six (46.15%) had received a second line of therapy before proceeding to alloSCT. Median time from diagnosis to alloSCT was 8 months (range 2–54 months), with a median follow-up after alloSCT of 10 months (range 2.7–28 months).

Five patients (38.5%) died after alloSCT. Their median Hematopoietic Cell Transplantation-specific Comorbidity Index (HCT-CI) score was 0 (range 0–4). All of them received reduced-intensity conditioning (Supplementary Table 3) [11]. Infection was the commonest cause of death (3 patients, 60%), and included COVID-19 pneumonia, *Saccharomyces cerevisiae* fungemia, and *Enterococus faecium* & *Escherichia coli* bacteremia. Patients who died after alloSCT had a higher incidence of grade 3–4 acute GVHD (20% vs. 12.5%). One patient (20%) died of colitis unrelated to graft-versus-host disease. None of the patients died of relapse. One patient (7.7%) had relapse at 7.5 months after alloSCT; he was in remission before alloSCT and was alive at last follow-up.

Median OS since diagnosis for patients undergoing alloSCT was 3.2 years. There was a trend towards inferior survival among patients undergoing alloSCT compared to those who did not have alloSCT (median OS 3.2 vs. 11.4 years, *P* = 0.06) (Fig. 1b). Overall survival did not differ among patients with a DDX41 VAF < 40% compared with VAF ≥ 40% (median OS 11.4 years *vs*. NA, *P* = 0.66).

The cumulative incidence of NRM was 0% after 30 days, 8.3% after 100 days and 44.8% at 1 year after alloSCT (Fig. 1c). NRM did not differ significantly between patients with AML and MDS, *P* = 0.69 (1-year NRM: 46.4% vs 40%). Cox-proportional hazard analysis with alloSCT as time dependent covariate showed that alloSCT adversely effected survival (hazard ratio 20.2, CI: 2.27–179.9, *P* = 0.007).

Our cohort had a predominantly male predisposition and a later age of onset in the late sixties. These findings are consistent with those reported in previous studies [6]. Most of the patients with *DDX41*-mutated MDS were classified as MDS-EB. Studies have shown *DDX41*-mutated MDS-EB to have favorable outcomes [4, 5].

Our study shows that patients with *DDX41*-mutated MDS/AML undergoing alloSCT had a trend towards lower survival. Although this finding did not reach statistical significance due to small sample size, patients undergoing alloSCT had a high rate of non-relapse mortality (1-year NRM 44.8%) despite low HCT-CI of 0–2 in most patients.

The post-transplant mortality was most commonly due to infections, highlighting the need to use lower intensity conditioning regimens and optimize infectious disease prophylaxis in these patients.

Due to the retrospective nature of our study, the comparison between alloSCT and non-alloSCT cohort is not without bias. However, patients undergoing alloSCT usually have better performance status and lesser comorbidities compared to patients who do not undergo alloSCT. Therefore, the inferior survival of the alloSCT cohort in our study is likely related to the alloSCT itself. It was recently shown that alloSCT did not improve survival in *DDX41*-mutated AML [12]. All five patients in our cohort who died after alloSCT were in complete remission before transplant and died most commonly of infections (Fig. 1a). While the majority of patients in our study underwent alloSCT due to high-risk disease, a subset of patients was deemed to have intermediate-risk disease, 40% of whom had non-relapse mortality, suggesting that alloSCT can potentially be deferred in these patients until disease progression or relapse.

In conclusion, this is the first study assessing post-transplant outcomes in patients with *DDX41*-mutated MDS/AML. Our study highlights a high post-transplant NRM in these patients, suggesting that alloSCT might need to be reserved for disease progression or relapse.

## Supplementary information

Supplemental tables

## Data Availability

Data may be obtained from the corresponding author upon reasonable request.
