# Supplementary material for: Outcomes of allogeneic transplant in patients with DDX41 mutated myelodysplastic syndrome and acute myeloid leukemia
Source: Bone Marrow Transplant. 2022 Aug 20;57(11):1716–8. doi: 10.1038/s41409-022-01776-6 (PMC9392432; doi:10.1038/s41409-022-01776-6)
Supplement: Supplementary file 1 — Supplemental tables [file 41409_2022_1776_MOESM1_ESM.pdf]

Supplemental Tables

Supplemental Table 1. Patient and disease characteristics stratified by alloSCT.

Characteristic		No alloSCT (n = 16)	alloSCT (n = 13)	*p*
Age at diagnosis (years)				<0.001
	Median	75	63	
	Range	50-82	54-70	
Gender, n(%)				0.364
	Female	6 (37.5%)	2 (15.4%)	
	Male	10 (62.5%)	11 (84.6%)	
Disease, n(%)				0.047
	MDS	14 (87.5%)	6 (46.2%)	
	AML	2 (12.5%)	7 (53.8%)	
MDS R-IPSS risk*, n = 20(%)				0.967
	Low	2 (14.3%)	1 (16.7%)	
	Intermediate	9 (64.3%)	4 (66.7%)	
	High	3 (21.4%)	1 (16.7%)	
AML ELN risk*, n = 9(%)				NA
	Favorable	0	0	
	Intermediate	2 (100%)	7 (100%)	
	Adverse	0	0	
Karyotype				NA
	Normal	13 (81.25%)	13 (100%)	
	Abnormal	3 (18.75%)	0	
	Complex	0	0	

Abbreviations: R-IPSS: Revised International Prognostic Scoring System, ELN: European Leukemia Network.
*Risk stratification at diagnosis.

Supplemental Table 2. Mutational profile of *DDX41* and other associated pathogenic mutations.

DDX41, pathogenic mutations	VAF range	Number of patients (%)*
c.1574G>A	7-19	9
c.3G>A	48-52	8
c.415_418dup	37-45	5
c.1589G>A	28-46	2
c.260del	7	1
c.1102C>T	49	1
c.121C>T	51	1
c.1588G>A	6	1
c.305_306del	46	1
c.434+1G>A	46	1
c.776A>G	51	1
c.931C>T	48	1
c.946_947del	47	1
***DDX41*, variance of unknown significance**	**VAF**	**Number of patients**
c.773C>T	46	2
c.710T>G	46	1
c.517G>A	50	1
c.959C>T	49	1
c.610C>T	40	1
c.622C>G	11	1
c.1032C>G	7	1
**Other associated genes with known pathogenic mutations**	**Gene**	**Number of patients**
	ASXL1	4
	DNMT3A	3
	EZH2	2
	JAK2	1
	NRAS	1
	PHF6	1
	SF3B1	1

Abbreviations: VAF: Variant Allele Frequency
*Total number is >29 due to few patients having >1 *DDX41* mutations.

Supplemental Table 3. Patient characteristics, transplant characteristics and transplant outcomes stratified by patient alive or dead at last follow-up.

Characteristic	Alive at last follow-up (n = 8)	Dead at last follow-up (n = 5)
Age at alloSCT (years)		
Median	66	63
Range	54-71	60-67
Median HCT-CI (Range)	1 (0 – 5)	0 (0 – 4)
Disease		
MDS	4 (50%)	2 (40%)
AML	4 (50%)	3 (60%)
Prior lines of treatment		
One	4 (50%)	3 (60%)
Two	4 (50%)	2 (40%)
Donor source		
Unrelated donor	4 (50%)	4 (80%)
Matched related donor	2 (25%)	1 (20%)
Haploidentical	2 (25%)	0
Conditioning regimen (intensity)		
Busulfan and fludarabine (MAC)	1 (12.5%)	0
Busulfan and fludarabine (RIC)	2 (25%)	2 (40%)
Fludarabine and melphalan (RIC)	3 (37.5)	3 (60%)
Fludarabine, cyclophosphamide and TBI (RIC)	2 (25%)	0
GVHD prophylaxis		
PTCY based	2 (25%)	2 (40%)
Tacrolimus-methotrexate	6 (75%)	3 (60%)
Acute GVHD		
Grade 2-4	4 (50%)	3 (60%)
Grade 3-4	1 (12.5%)	1 (20%)
Chronic GVHD		
Mild	0	0
Moderate	3 (37.5%)	1 (20%)
Severe	0	1 (20%)
Relapse	1 (12.5%)	0

Abbreviations: MAC: Myeloablative conditioning, PTCY: Post-transplant cyclophosphamide, RIC: Reduced intensity conditioning, TBI: Total body irradiation.
